# Effect of Preheating and Precooling on the Flexural Strength and Modulus of Elasticity of Nanohybrid and Silorane-based Composite

**Published:** 2015-09

**Authors:** Farahnaz Sharafeddin, Mehran Motamedi, Zahra Fattah

**Affiliations:** a Biomaterial Research Center, Dept. of Operative Dentistry, School of Dentistry, Shiraz University of Medical Science, Shiraz, Iran.; b Dept. of Operative Dentistry, School of Dentistry, Shiraz University of Medical Science, Shiraz, Iran.; c Post Graduate Student, Student Research Committee, Dept. of Operative Dentistry, School of Dentistry, Shiraz University of Medical Science, Shiraz, Iran.

**Keywords:** Nanohybrid resin composite, Silorane-based resin composite, Flexural strength, Flexural modulus, Preheating, Precooling

## Abstract

**Statement of the Problem:**

Composite resin may be used in different temperatures; it is crucial to determine the effect of temperature on mechanical properties of nanohybrid and silorane-based composite.

**Purpose:**

This *in vitro* study compared the flexural strength and modulus of elasticity of nanohybrid and silorane-based resin composite, at 4˚C, room temperature (25˚C), and 45˚C.

**Materials and Method:**

In this experimental study, 60 specimens were prepared in a metal split mold (2×2×25mm). Two different resin composites, Filtek Z250 XT (3M/ ESPE) and Filtek P90 (3M/ESPE), were evaluated. The material were inserted into split molds at room temperature, 4˚C or 45˚C and cured with LED (1200 mW/cm^2^) for 20 seconds in four points (n=10). Then, a three-point bending test was performed using a universal testing machine at a crosshead speed of 0.5 mm/min for measuring the flexural strength and flexural modulus of samples. The data were analyzed by the two-way ANOVA and Tukey test (*p*< 0.05).

**Results:**

The mean highest flexural strength was observed at 45˚C, showing statistically significant difference with flexural strength at 4˚C (*p*= 0.0001) and 25˚C (*p*= 0.003) regardless of the type of resin composite. The flexural modulus at 45˚C was highest, showing the statistically significant difference with flexural modulus at 4˚C (*p*= 0.0001) and 25˚C (*p*= 0.002). The flexural modulus was statistically different between nanohybrid and silorane-based resin composite (*p*= 0.01) in 25˚C and 45˚C, but there were no statistically significant differences between flexural strength of Filtek Z250 XT and Filtek P90 regardless of the temperatures (*p*= 0.062).

**Conclusion:**

Preheating the resin composite at 45˚C improves flexural strength and modulus of nanohybrid and silorane-based resin composite. However, flexural strength and modulus of the tested materials were not affected by precooling. The flexural modulus of nanohybrid resin composite was significantly higher than silorane-based resin composite in 25˚C and 45˚C temperatures.

## Introduction

Increasing the temperature of restorative material prior to placing in the cavity has recently gained popularity among dental practitioners.(1) Generally, preheating causes a reducing viscosity which promotes improvement of marginal adaptation, enhanced handling properties(1) increased polymerization rate, degree of conversion,(2) and improved mechanical and physical properties.(3)

It has been reported that pre-heating composite has same or higher degree of conversion as composite cured at room temperature even if the light-curing time was reduced as much as %75,(4) reducing the photo-curing time, which in turn, increases efficiency and decreases the chair time. A previous study showed that cooling before light-curing did not affect the hardness of resin composite after polymerization.(5) Moreover, it was observed that precooling decreases the shrinkage of composite resins.(6) Therefore, manufactures recommend keeping the composite syringes inside the refrigerator. Therefore, it seems the effect of precooling on mechanical properties of resin composite should be studied.

A few new material developments, such as nanohybrid and silorane-based resin composite have been studied during the last decade. Nanohybrid composites have been produced by adding Nano sized particle (5-100nm)(7) in the microhybrid resin composites. Silorane- based resin composites have a new silorane monomer that is the combination of oxiran and siloxan.(8) Their mechanical properties are the same as, or even better than, the methacrylate-based composites. Two important advantages of silorane-based composite are increased hydrophobicity due to the presence of siloxan and low polymerization shrinkage due to the ring-opening siloxan.(9)

To the best of authors’ knowledge, the effect of preheating on the mechanical properties of nanohybrid resin composites and comparing it with microfilled resin composites has already been studied, while the effect of precooling has not yet been examined. Also, the effect of temperature on the mechanical properties of silorane-based resin composite has not been studied yet. The flexural strength and modulus are fundamental mechanical properties for brittle materials,(10) hence, it seems that it is of utmost importance to examine the effect of different temperatures on the flexural strength and modulus of resin composite.

The aim of this *in vitro* study was to compare the flexural strength and modulus of precooling, preheating and room temperature on silorane-based and nanohybrid resin composite. The null hypotheses were as the flexural strengths and modulus would not be affected by composite-based materials and also the flexural strengths and modulus would not be affected by temperature of resin composite.

## Materials and Method

In this experimental study, 60 specimens of two resin composite material in 6 groups (n=10) were prepared. A nanohybrid composite Filtek Z250 XT (3M ESPE USA) and a silorane-based resin composite Filtek P90 (3M ESPE USA) were used (Table 1). An incubator was used to obtain the temperature of 25˚C and 45˚C for the composite resins. Composite syringes were placed in the incubator for at least 15 minutes so that the temperature rose to 25˚C and 45˚C.(11) Composite syringes were placed in the refrigerator for at least 30 minutes to stabilize the cooled temperature (4˚C).(11)

**Table 1:** The brand names, manufacture, chemical composition and batch numbers of the materials used in this study.

**Brand/ Manufacturer**	**Batch Number**	**Chemical composition**
Filtek Z250 XT (shade A2),/ 3M EPSE, St Paul, MN, USA	N431404	BIS-GMA, UDMA, BIS-EMA, TEGDMA. Silica particle 20 nm and Zirconia/Silica particle 10-0.1 microns (%67.8 by volume)
Filtek P90 (shade A2),/ 3M EPSE, St Paul, MN, USA	N384451	3,4-epoxycyclohexylrthhylcyclo- polymethylsiloxane,bis-3,4-epoxycyclo- hexy lethylphenyl methylsilane. Camphorquinone, stabilizer and pigments. Quartz/ yttrium fluoride (53%by volume)

**Figure 1:**
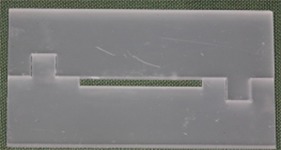
Split molds

The materials were inserted into split rectangular models with a slot in central part of the mold with the dimensions specified by the ISO 4049/2000 specification (25×2×2 mm) (Figure 1).

The mold was positioned over a glass slide and a Mylar strip and the resin composite was inserted as a single increment at room temperature (25˚C), precooled (4˚C) and preheat (45˚C). The total insertion time from the material removal from the syringe to the insertion of the composite resin in the mold in one increment was approximately 40 seconds. Then Mylar strip was positioned over the composite and pressed against with a glass slide, so the excess of material in the corner was carefully removed before polymerization. Then, the specimens were cured with LED (Demetron; Kerr, Switzerland) with 1200mW/cm(2)intensity for 20 seconds at four points because the diameter of the tip of light guide was 8mm and the length of mold was 25mm. All the specimens were stored in water for 24 hours in the room temperature. After the removal of the specimens from the split molds, a three-point bending test was performed using a universal testing machine (ZWICK/ ROELL ZO20, Germany) at a crosshead speed of 0.5 mm/min. The maximum loads were obtained and flexural strength (σ) was calculated in mega Pascal (MPa) by using the following equation:

$$\sigma =\;3FL/\;2BH{\;}^{2}\;\;\;$$

$$E=FL{\;}^{3}/4BH{\;}^{3}d\;$$

where F is the maximum load (N); L is the length of the specimen (mm); B is the width of the specimen (mm); H is the height of the specimen (mm) and d is the deflection (mm) corresponding to the load F.

The data were analyzed by one-way analysis of variance (ANOVA) and Tukey HSD test (*p*< 0.05).

## Result

The results were subjected to the one-way analysis of variance (ANOVA) and the differences between the materials and temperatures were assessed with Tukey test to determine the effect of temperatures on the materials with the significance level set at *p*< 0.05.

Table 2 summarizes the mean values and standard deviations of flexural strength of the tested groups. The one-way ANOVA showed that there were no statistically significant differences (*p*= 0.062) between flexural strength of Filtek Z250 XT and Filtek P90 regardless of the temperature differences. Given the composite resin temperatures of 4˚C, 25˚C and 45˚C, there were significant differences in flexural strength (*p*= 0.0001) regardless of the type of composite resin material that have been used.

**Table 2:** Mean values and standard deviations of Flexural strength the tested groups.

**Temperature** **Composite**	**4˚C**		**25˚C**		**45˚C**	
	**Mean**	**SD**	**Mean**	**SD**	**Mean**	**SD**
Filtek Z250 XT	111.70Aa^a^	6.70	118.30Aa	13.54	134.40Ba	11.00
Filtek P90	104.71Aa	10.00	115.37Aa	12.395	126.30Ba	17.06

^a^ Means followed by the same letter indicates no significant statistical difference in the row and the same lowercase letter indicates no significant statistical difference in the column (p> 0.05)

There was a statistically significant difference between the mean highest flexural strength at 45˚C and that at 4˚C (*p*= 0.0001) and also between the flexural strength at 45˚C and 25˚C (*p*= 0.003). However, there was no statistically difference between the flexural strength at 4˚C and 25˚ C (*p*= 0.074).

**Figure 2:**
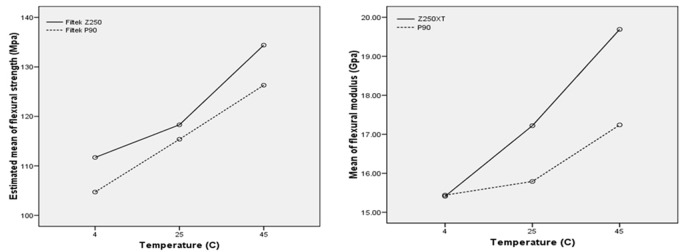
Mean of flexural strength and modulus of the resin composites

While there were no statistically significant differences between two resin composites in flexural strength value, there were statistically significant differences (*p*= 0.01) between two composite resin in flexural modulus in 25˚C (*p*= 0.01) and 45˚C (*p*= 0.01) (Table 3) but there was no statistically significant differences between two resin composites in 4˚C in flexural modulus. The flexural modulus and strength of tested group had the similar behavior in three different temperatures regardless of the type of resin composite (Figure 2).

**Table 3:** Mean values and standard deviations of Flexural Modulus of the tested groups.

**Temperature** **Composite**	**4˚C**		**25˚C**		**45˚C**	
	**Mean**	**SD**	**Mean**	**SD**	**Mean**	**SD**
Filtek Z250 XT	15.440Aa	1.75	17.220Aa	1.37	19.690Ba	2.01
Filtek P90	15.410Aa	1.63	15.790Ab	2.56	17.240Bb	1.63

^a^ Means followed by the same uppercase letter indicates no significant statistical difference in the row and the same lowercase letter indicates no significant statistical difference in the column (*p*> 0.05)

## Discussion

In this study, the flexural strength and flexural modulus were assessing to compare the functions of nanohybrid and silorane-based resin composites in three different temperatures. Fractures within the body of restorations and at the margins have been cited as a major problem in the failure of posterior resin composites.(10) The fracture-related properties of resin based composites, such as fracture resistance, elasticity and the marginal degradation of materials under stress have generally been evaluated by determining the material parameters such as flexural strength and flexural modulus.(12-14) Although the findings cannot be extrapolated to the clinical behavior without considering other factors such as flaw distribution(15) and the structural reliability of the material,(16) the *in vitro* three-point bending flexural test is recommended by the ISO 4049/2000(17) specification for polymer-based materials and is widely used for comparative purposes.(18)

The present study showed that the flexural strengths of the methacrylate-based nanohybrid and silorane-based composite resins presented no statistically significant difference (*p*= 0.062) at 4˚C, 25˚C and 45˚C, therefore, the first hypothesis was partially accepted. Uctasli(19) *et al.* have examined the effect of pre-heating and room temperature on the nanohybrid and microfilled resin composite and found that there were no statistically difference between the flexural strengths of the tested materials. Also in current study, the flexural strength of nanohybrid and silorane-based resin composites were not statistically different. A few studies have examined the effect of preheating and precooling on the nanohybrid resin composite, but to the best of our knowledge, the effect of temperature on the silorane-based resin composites has not been studied yet.

The findings of the study showed that the flexural strengths of nanohybrid and silorane-based resin composite were higher at temperature of 45˚C, since heating composite resins prior to placement increases the monomer conversion and polymerization rate.(20) With increased paste temperature, free radicals and propagating polymer chains become more mobile as a result of decreased paste viscosity which results in a more complete polymerization reaction (more double-bond formation) and greater cross-linking.(20) However, the flexural strengths of resin composite were not significantly different at temperatures of 4˚C and 25˚C. This justifies that removing the resin composite syringe from the refrigerator immediately before its use will not have any adverse effect on the flexural strength of composite resin. Froes-Salgado(21) *et al.* conducted a study that evaluated the effect of composite pre-polymerization temperature on flexural strength of a nanofilled composite (Filtek Z350, 3M/ESPE) and found that pre-heating the composite prior to light polymerization did not alter the flexural strength. In our study, however, preheating (45˚C) improved the flexural strength of nanohybrid and silorane-based composite resin.

Filler content, filler size, and distribution of filler would influence the physical and mechanical properties of resin composites.(22) It has been reported that the filler volume and filler load level of resin composite have a strong correlation with material strength and elastic modulus.(19) Kim *et al.*(23) have reported that the mechanical properties of resin composite were related to their filler content. So, resin composites with higher filler loading exhibit higher flexural strength and modulus. Filtek P90 composite is filled with a combination of fine quartz particles and radiopaque yttrium fluoride and is classified as a microhybrid resin-composite. The concentration of filler particles in this composite is 53% by volume. The filler in Filtek Z250XT is zirconia/silica in a concentration of 67.8% by volume and is classified as nanohybrid resin composite. In the present study, resin composite with the higher filler loading (Filtek Z250XT) has exhibited the higher flexural strength and the composite with lower filler content (Filtek P90) has shown lower flexural strength. This result is in agreement with previous findings.

A tooth restoration material should mimic mechanical and physical characteristics of dentin and enamel.(24-25) Xu *et al.*(26) measured the elastic modulus of human enamel and dentin and obtained a mean value of 94 Gpa and 19 Gpa for enamel and dentin respectively. Flexural modulus of nanohybrid and silorane-based composite resin in all tested temperatures were different from those of enamel. However, the present study showed that nanohybrid resin composite (Filtek Z250XT) had mean flexural modulus (17.44) similar to the flexural modulus of dentin that can be effective in longevity of restorations.

While flexural strengths of nanohybrid and silorane-based resin composites were not statistically different, there were statistically significant differences between the flexural modulus of nanohybrid and silorane-based resin composites in 25c˚ and 45c˚ (*p*= 0.01). The nanohybrid resin composite (Filtek Z250XT) had higher flexural values than silorane-based resin composite (Filtek P90). Flexural modulus had similar behavior to flexural strength regardless of the type of resin composite in all tested temperatures. It was observed that flexural modulus of both resin composites has been improved by preheating temperature (45˚C) but the flexural modulus was not significantly different at temperature of 4˚C and 25˚C.

Beun *et al.*(27) reported that the nanohybrid resin composite had higher flexural modulus while the universal hybrid resin composite had higher flexural strength. In another study, Uctasli *et al.*(19) concluded that no significant difference was found between flexural strength of microhybrid (Filtek Z250) and nanohybrid (Grandio) resin composite while flexural modulus of nanohybrid resin composite was statistically higher than microhybrid resin composite. In the present study, nanohybrid resin composite (Filtek Z250XT) exhibited higher flexural strength and modulus than silorane-based resin composite (Filtek P90). This might be due to the different filler loading of the tested composite. Resin composite with the lowest filler content (Filtek P90) presented lower modulus in comparison with Filtek Z250X. However, no study has compared the effect of filler content on the flexural strength of silorane-based resin composite with nanohybrid resin composite. The findings of our study, along with some preceding ones, showed that different filler volume fractions and filler load levels of the resin composite would result in different elastic modulus of materials.

Although these are *in vitro* results, they are of significance because these factors cannot easily be quantitatively determined *in vivo*. Nevertheless, further clinical studies are necessary to confirm these results.

## Conclusion

It can be concluded the precooling (4˚C) and room temperatures (25˚C) did not have any effects on the flexural strength and modulus but preheating the resin composite at 45˚C improved the flexural strength and flexural modulus of nanohybrid and silorane-based resin composites. There were also significant differences between flexural modulus of the two resin composites in two different temperatures (4c˚and 45c˚) and the mean flexural modulus of silorane-based resin composite (Filtek P90) was significantly higher than that of nanohybrid resin composite (Filtek Z250XT). 

## References

[1] Tantbirojn D, Chongvisal S, Augustson DG, Versluis A (2011). Hardness and postgel shrinkage of preheated composites. Quintessence Int.

[2] Blalock JS, Holmes RG, Rueggeberg FA (2006). Effect of temperature on unpolymerized composite resin film thickness. J Prosthet Dent.

[3] Asmussen E, Peutzfeldt A (2001). Influence of pulse-delay curing on softening of polymer structures. J Dent Res.

[4] Daronch M, Rueggeberg FA, De Goes MF (2005). Monomer conversion of pre-heated composite. J Dent Res.

[5] Osternack FH, Caldas DB, Rached RN, Vieira S, Platt JA, Almeida JB (2009). Impact of refrigeration on the surface hardness of hybrid and microfilled composite resins. Braz Dent J.

[6] Walter R, Swift EJ Jr, Sheikh H, Ferracane JL (2009). Effects of temperature on composite resin shrinkage. Quintessence Int.

[7] Moszner N, Klapdohr S (2004). Nanotechnology for dental composites. Int J Nanotechnol.

[8] Ilie N, Hickel R (2009). Macro-, micro- and nano-mechanical investigations on silorane and methacrylate-based composites. Dent Mater.

[9] D'Alpino PH, Bechtold J, dos Santos PJ, Alonso RC, Di Hipólito V, Silikas N (2011). Methacrylate- and silorane-based composite restorations: hardness, depth of cure and inter facial gap formation as a function of the energy dose. Dent Mater.

[10] Roulet JF (1988). The problems associated with substituting composite resins for amalgam: a status report on posterior composites. J Dent.

[11] Woolum JA, Berry TG, Wilson DE, Hatch R (2008). Benefits of preheating resin composite before placement. Gen Dent.

[12] Sakagughi RL, Powers JM Craig,s (2012). Restorative dental material.

[13] Sharafeddin F, Tondari A, Alavi AA (2013). The Effect of Adding Glass and Polyethylene Fibers on Flexural Strength of Three Types of Glass-Ionomer Cements. Res J of Biologic Scien.

[14] Sharafeddin F, Alavi A, Talei Z (2013). Flexural strength of glass and polyethylene fiber combined with three different composites. J Dent (Shiraz).

[15] Loughran GM, Versluis A, Douglas WH (2005). Evaluation of sub-critical fatigue crack propagation in a restorative composite. Dent Mater.

[16] Bona AD, Anusavice KJ, DeHoff PH (2003). Weibull analysis and flexural strength of hot-pressed core and veneered ceramic structures. Dent Mater.

[17] ISO 4049:2000- Dentistry -- Polymer-based filling, restorative and luting materials: ISo; 2000.

[18] Chung SM, Yap AU, Chandra SP, Lim CT (2004). Flexural strength of dental composite restoratives: comparison of biaxial and three-point bendingtest. J Biomed Mater Res B Appl Biomater.

[19] Uctasli MB, Arisu HD, Lasilla LV, Valittu PK (2008). Effect of preheating on the mechanical properties of resin composites. Eur J Dent.

[20] Choudhary N, Kamat S, Mangala T, Thomas M (2011). Effect of pre-heating composite resin on gap formation at three different temperatures. J Conserv Dent.

[21] Fróes-Salgado NR, Silva LM, Kawano Y, Francci C, Reis A, Loguercio AD (2010). Composite pre-heating: effects on marginal adaptation, degree of conversion and mechanical properties. Dent Mater.

[22] Sharafeddin F, Sharifi E (2013). The effect of microwave/ laboratory light source postcuring technique and wet-aging on microhardness of composite resin. Dent Res J (Isfahan).

[23] Kim KH, Ong JL, Okuno O (2002). The effect of filler loading and morphology on the mechanical properties of contemporary composites. J Prosthet Dent.

[24] Sabbagh J, Vreven J, Leloup G (2002). Dynamic and static moduli of elasticity of resin-based materials. Dent Mater.

[25] Sharafeddin F, Bahrani S (2011). Load bearing capacity of fragmented incisal edge restored with two different positions of fiber reinforced composite restoration. J Dent (Shiraz).

[26] Xu HH, Smith DT, Jahanmir S, Romberg E, Kelly JR, Thompson VP (1998). Indentation damage and mechanical properties of human enamel and dentin. J Dent Res.

[27] Beun S, Glorieux T, Devaux J, Vreven J, Leloup G (2007). Characterization of nanofilled compared to universal and microfilled composites. Dent Mater.

